# Symptoms of Emotional, Behavioral, and Social Difficulties in the Danish Population of Children and Adolescents with Type 1 Diabetes – Results of a National Survey

**DOI:** 10.1371/journal.pone.0097543

**Published:** 2014-05-19

**Authors:** Lene J. Kristensen, Niels H. Birkebaek, Anne H. Mose, Lena Hohwü, Mikael Thastum

**Affiliations:** 1 Department of Psychology and Behavioural Sciences, Aarhus University, Aarhus, Denmark; 2 Department of Pediatrics, Aarhus University Hospital, Aarhus N., Denmark; 3 Department of Public Health, Aarhus University, Aarhus, Denmark; McGill University, Canada

## Abstract

**Objective:**

To assess the prevalence of psychological difficulties in Danish children and adolescents with type 1 diabetes using both child/adolescent and caregiver reports, and to investigate associations between these symptoms and metabolic control, adherence, and quality of life.

**Research Design and Method:**

A total of 786 children and adolescents (8–17 years) recruited through the Danish Registry of Childhood Diabetes completed subscales of the Beck's Youth Inventories (BYI-Y), while 910 caregivers completed the Strength and Difficulties Questionnaire (SDQ). The participants also completed questionnaires assessing adherence and quality of life. BYI-Y and SDQ responses were compared with results from normative samples.

**Results:**

Children with diabetes generally reported a lower level of symptoms of depression and anxiety, while older adolescents in most cases were comparable to the normative samples. However, the numbers of patients with elevated scores were similar to normative groups, especially regarding the proportion of participants with ‘Extremely elevated’ scores. Caregivers of children and adolescents with diabetes generally reported the prevalence of elevated scores on the SDQ to exceed the prevalence observed in the norm sample – particularly with regard to older boys. Both BYI-Y and SDQ responses were significantly correlated with HbA_1c_, adherence, and quality of life.

**Conclusions:**

This study finds Danish children and adolescents with diabetes to report lower or comparable levels of emotional difficulties compared to norms, while caregiver reports are less positive. The results therefore support the value of a multi-informant approach to the assessment of symptoms of psychological difficulty in girls and boys with diabetes.

## Introduction

Navigating through the many naturally occurring challenges of childhood and puberty while facing the extra burden of living with a chronic disease can increase the risk of experiencing psychological difficulties [1], [2]. A number of studies have assessed the prevalence of emotional and behavioral problems in children and adolescents with type 1 diabetes. Particularly in relation to symptoms of depression [3]–[5], symptoms of anxiety [6], and to a lesser extent externalizing behavior problems [7]–[9].

The prevalence of these difficulties varies greatly among studies. Some studies have found children and adolescents with diabetes to be similar to control groups of children and adolescents without diabetes regarding the prevalence of psychological difficulties [10], [11]; however, in the majority of studies, especially those regarding symptoms of depression in this patient group, the prevalence appears to be somewhat increased [12], [13], particularly among adolescent girls with diabetes compared with age-matched comparative samples [14]. A recent review by Reynolds and Helgeson [15] confirms that children with diabetes are at slightly elevated risk of psychological difficulties compared with comparison groups, although the effect sizes of these differences are smaller in more recent studies.

Although psychological difficulties among youths with diabetes have been the subject of a number of empirical studies, the consequences of these emotional and social problems are less clear. A few studies have shown symptoms of depression to be associated with an increased risk of severe hypoglycemia [16], being hospitalized with diabetes complication [17], and poorer quality of life [13]. Some studies have found depression symptoms, anxiety, and behavior problems to be associated with suboptimal metabolic control [6], [18], [19], and less-than-optimal adherence [20], [21], although other studies have not been able to verify these connections [9], [12].

The psychological well-being of the Danish pediatric type 1 diabetes population has received very little research attention, nor has the possible consequence of emotional and behavioral difficulties on the diabetes treatment of this group been investigated. Socio-economic and cultural factors, such as low poverty levels, high living standards, and equal access to public health care, and therefore a lower economic burden on the family, make it plausible that this Danish population differs in some respects from other international study populations.

While aiming at evaluating symptoms of depression, anxiety and behavioral problems in Danish children and adolescents with type 1 diabetes, this study also sought to overcome some of the shortcomings of previous studies by including a large population sample with a wide age range, and by containing both child and caregiver assessments of psychological difficulties.

The purpose of the present study is to assess the prevalence of emotional and behavioral difficulties in Danish children and adolescents with type 1 diabetes by comparing both child/adolescent and caregiver reports to results from normative samples, and to investigate associations between these symptoms and metabolic control, adherence, and quality of life in the pediatric diabetes sample. Though empirical findings regarding the possible relationship between psychological difficulties and metabolic control, adherence, and quality of life are somewhat inconsistent, we expected symptoms of depression, anxiety, and behavior problems to be associated with suboptimal metabolic control, poorer adherence to treatment, and quality of life.

## Research Design and Methods

### Ethics Statement

The department where the study was conducted did not have an institutional review board, so in accordance with Danish procedure the regional ethic committees (De Videnskabsetiske Komitéer for Region Midtjylland) were consulted. In keeping with the regulations of the Committees, questionnaire-based studies do not have to be approved; however, a study protocol was provided to the Committees who confirmed, that although participating children and adolescents were asked to submit a small blood sample (comparable to their daily blood glucose testings), no biological samples were collected with the intent of establishing a research bio-bank, therefore the project was not encompassed by the term ‘Bio-medical research’, and as such not eligible for Committee review and approval. The project was registered at The Danish Data Protection Agency (Ref no. 2013-41-1528). Written informed consent was obtained from all participants (either through the online version of the questionnaires, or on paper), including caregivers consenting to the participation of their child/adolescent. All families were given thorough written information that their participation was voluntary and anonymous to all other than the first author, and that their consent could at all times be withdrawn, just as refusal of participation did not in any way influence the diabetes treatment that the child/adolescent was receiving.

### Design

The study was conducted in collaboration with the Danish Society for Diabetes in Childhood and Adolescent, who administers the Danish Childhood Diabetes Registry (the Registry).

The Registry was the principal source of identification of eligible participants for the survey. Included in the Danish Registry of Childhood Diabetes are nearly all children and adolescents, who are treated at the Danish diabetes centers as well as all newly diagnosed patients between the ages of 0 and 15. The Registry is based on patient data provided annually by all pediatric hospital units treating children and adolescents with diabetes. Data regarding the diabetes treatment and status, HbA_1c_-level, physiological variables (height, weight, and pubertal status) and episodes of hypoglycemia and ketoacidosis of each child/adolescent is recorded.

Based on patient information from the Registry 1691 families with a child/adolescent with diabetes aged 4–17 years were invited to participate in the present survey. Excluded were only those families (*n* = 258) who were registered as either unwilling to participate in scientific research, had a protected address, or were no longer residing at the address registered in the Danish Civil Registration System, from which all participant addresses were collected.

All families eligible for participation received a written invitation by post, asking them to participate in a national web survey investigating well-being and quality of life in the pediatric diabetes population. They were also given the option of completing a paper version of the questionnaire, if they should so prefer.

The caregiver primarily involved with the diabetes-related care of the child/adolescent was requested to complete the survey. Adolescents and children 8 years and older were also requested to complete an individual version of the survey.

All families were asked to send in a small blood sample from the child; these samples were analyzed centrally at Glostrup Hospital, which was responsible for all HbA_1c_ measures included in the Registry at the time.

### Measures

Socio-demographic and clinical data were provided by participants and by the Registry.

The depression (BDI-Y) and anxiety (BAI-Y) subscales of the The Beck's Youth Inventories – Second edition (BYI-II) [22] were completed by children and adolescents aged 8–17 years. Each subscale consists of 20 questions, and is scored by calculating a total score ranging from 0 to 60, with higher scores indicating more symptoms of depression or anxiety. No caregiver version of the questionnaire exists. Responses from the diabetes sample were compared with self-reported data from a Danish sample comprised of 1444 children and adolescents aged 7–17 years from which the Danish norms for the BYI-II were calculated. The sample is described in further details in the Danish manual of the BYI-II [22]. A study by Thastum et al. among 8–14 year old Danish children showed that the Danish BYI has adequate reliability and test-retest stability; significant differences have been observed between normative and clinical groups regarding both the depression and anxiety subscales [23].

Caregivers of children/adolescents aged 4–16 years completed the Strength and Difficulties Questionnaire (SDQ). The SDQ is a brief, 25-item behavioral screening instrument consisting of five separate 5-item subscales that generate scores for Emotional Symptoms, Conduct Problems, Hyperactivity-Inattention, Peer Problems, and Prosocial Behavior, respectively [24]. The SDQ has been widely used, and satisfactory psychometric properties of the instrument, including reliability and validity, have been established in several studies [25]–[27]. Children and adolescents that were part of a large, Danish study were used as the normative sample for the purpose of comparison. A total of 3200 children living in Denmark aged 2–17 years (100 boys and 100 girls from each age group) were randomly selected to participate in the third wave of the “Health and welfare among children and young people in the Nordic countries 2011” (NordChild) data collection. Data for the NordChild survey was previously collected in 1984 and 1996 in all Nordic countries: Finland, Iceland, Norway, Sweden, and Denmark [28], [29]. The Danish Board of Health oversaw the random selection of children in the Danish part of NordChild 2011. As part of the study, 1655 Danish parents (51.7%) and their children completed the SDQ. The SDQ responses of 1375 of the parents from the norm sample that had children aged 4–16 years were compared with responses from the caregivers of the diabetes survey. The NordChild survey has been described in further details by Hohwü et al. [30].

The Adherence in Diabetes Questionnaire (ADQ) [31] was completed by all caregivers and children/adolescents aged 10–17 years. The ADQ was used to assess the level of adherence behavior related to diabetes care. Two versions of the ADQ were developed for the purpose of this study: one, for children and adolescents on conventional treatment, consisting of 19 items for children and their parents; the other, for children and adolescents on an insulin pump, consisting of 17 questions for children and their parents. Both versions exhibited good psychometric properties [31]. The questionnaires are scored by calculating the mean of all items. Higher scores indicate better adherence.

The Pediatric Quality of Life Inventory −4.0 Generic Core Scales (PedsQL – Generic) was administered to all caregivers and children/adolescents in this study as a measure of quality of life. The PedsQL – Generic is a 23-item questionnaire comprising subscales measuring physical and psychosocial (emotional, social, and school functioning) aspects of quality of life [32]. The reliability and validity of the PedsQL - Generic were established previously [33]. It is scored by calculating mean scores for each subscale, as well as for the total score. Higher scores indicate better health-related quality of life of the child/adolescent.

### Data Analysis

All statistical analyses were conducted using SPSS version 19. As is common with questionnaire responses from scales measuring psychosocial problems, the data reported here was not normally distributed. However, because of the large samples, parametric statistics were chosen as the method of data analysis when looking at the entire sample, while non-parametric statistics were used when looking at smaller subgroups of participants divided according to age and sex.

The age group divisions used in this study was chosen not only to fit the age group requirements for comparing BDI-Y and BAI-Y results in accordance with the Danish manual of the BYI-II [22], but also to enable comparison across measures. Therefore participants were divided into 5 age groups: 1) 4–7 year old; 2) 8–10 years old; 3) 11–14 years old; 4) 15–16 years old; and 5) 17 years old. For group 1 only caregiver assessment regarding psychosocial strength and difficulties were collected (SDQ), and for group 5 only self-report data regarding emotional problems were included (BDI-Y and BAI-Y). For the remaining groups both self-report data and caregiver reports were collected (BDI-Y, BAI-Y, and SDQ).

Independent-Samples *t*-tests were used to compare participating children and adolescents with non-participants, and also to assess between-group differences regarding BDI-Y, BAI-Y, and SDQ Total Difficulties scores based on the HbA_1c_ level of the child. Cohen's *d*'s were calculated to assess the effect size of the mean differences. Cohen's conventions for evaluating effect sizes were applied, where a *d* of.20 represents a weak or small association; a *d* of.50 is considered a moderate correlation; and a *d*.80 or larger represents a strong or large effect size [34].

A paired-samples t-test was conducted to assess possible differences between HbA_1c_ samples provided by the participants for the present study, and those HbA_1c_ values that were obtained from the Danish Childhood Diabetes Registry.

Mann-Whitney *U* tests were used to evaluate between-group differences in BDI-Y, BAI-Y, and SDQ scores for subgroups based on age and sex. The accompanying *z*-scores were used to calculate the effect size estimate, *r*, where a *r* = .10 represents a small effect, *r* = .30 represents a medium effect, and *r* = .50 represents a large effect [34].

Kruskal-Wallis tests were conducted to assess between-group differences for the entire diabetes sample based on the family structure/living situation of the child/adolescent, followed by Mann-Whitney *U* tests to identify the particular groups that differed significantly.

To compare the number of participants in the diabetes sample with elevated scores on the BDI-Y, and BAI-Y to the normative samples, self-report scores were categorized as either ‘Normal’, ‘Slightly elevated’, ‘Moderately elevated’, and ‘Extremely elevated’ as described in the Danish manual of the BYI-II [22]. Frequency analyses were conducted to assess percentage of participants and norm with elevated scores.

Following Goodman's recommendations [24] with approximately 80% of a norm population being in the ‘Normal’ range, 10% in the ‘Borderline’ range, and the 10% of the population with the highest scores being in the ‘Abnormal’ or ‘Clinical’ range, frequency distributions of norm parent scores were used to establish ‘Borderline’ and ‘Clinical’ cut-offs, and then applying these cut-offs to the diabetes sample. *Z* test calculations for comparing two population proportions were then used to evaluate the significance of inter-sample differences. Pearson's bivariate correlations were used to assess associations between symptoms of depression, anxiety, and behavioral problems, and the outcome variables of adherence, metabolic control, and quality of life, as well as socio-demographic, and treatment-related variables.

## Results

### Participant characteristics

Of the 1691 families who were invited to participate, a total of 1034 (61.1%) families provided data for the current study. 786 (51.9%) children and adolescents completed the BDI-Y and the BAI-Y; 910 (53.8%) caregivers completed the SDQ. Please refer to Table 1 for sample characteristics.

**Table 1:** Sample characteristics.

Number of participating caregivers/children and adolescents	910/786
Age of responding child/adolescent (years)	13.4±2.6
Sex of child	51.5% girls
Number of participating families that provided a blood sample from child/adolescent with diabetes	908
Mean HbA_1c_ level of child/adolescent from participating families based on submitted blood samples	8.04±1.13
Mean HbA_1c_ level of child/adolescent from participating families based on registry data	8.06±1.14
Diabetes duration (years)	5.24±3.28
Using insulin pump	42.8% (443 of 1034 participants)
Living situation of children/adolescents with diabetes	77.4% of children/adolescents live with both biological parents
	3.5% of children/adolescents take turn living with both divorced parents
	8.9% of children/adolescents live with single parent
	6.7% of children/adolescents live with one biological parent and his/her new partner
	2.1% of children/adolescents live at continuation school (boarding school for 14–18 year old students)
	1.2% Child/adolescent lives in foster care or at treatment facility
	0.1% has left home to live by himself/herself
Sex of responding caregiver	82.7% women

Data are reported as means ±SD unless otherwise indicated.

The sex distribution among children and adolescents from participating families resembled that of the non-participants (51.5% girls). However, non-participants differed significantly as a group in that they were slightly older (*M* = 13.2 years, *SD* = 3.2) than the children of participating families (*M* = 12.3 years, *SD* = 3.7, *p*<.001, *d* = 0.2), and had been diagnosed somewhat longer (*M* = 6.2 years, *SD* = 3.6) than participants (5.2 years, *SD* = 3.3, *p*<.001, *d* = 0.3). Based on Registry data, HbA_1c_ levels were significantly lower among participants (*M* = 8.05, *SD* = 1.17) than non-participants (*M* = 8.71, *SD* = 1.44, *p*<.0001, *d* = 0.5); however, the effect sizes for these differences were all relatively small to moderate.

As a paired-samples t-test revealed no statistically significant differences between HbA_1c_ scores provided for the study (*M* = 8.04, *SD* = 1.13), and those obtained from the Registry for the same participants (*M* = 8.05, *SD* = 1.17, *t*(895)  = −0.39, *p* = 0.70), HbA_1c_ level from the Registry were used for analyses, if participants did not provide a blood sample for the study.

### Symptoms of depression, anxiety, and social and emotional difficulties

The median BDI-Y, BAI-Y, and SDQ scores of the participating children and adolescents were compared with national norms (Table 2). For both sexes, participants with diabetes had significantly lower median BDI-Y scores than Danish age-matched children and adolescents, with effect sizes between 0.15 and 0.39. Only the 15- to 16-year-old boys, and 17 year old boys and girls had BDI-Y scores that were comparable to the norm group. A similar pattern emerged with respect to median BAI-Y scores, where nearly all age and sex group of participants with diabetes reported lower levels of symptoms of anxiety than the norm groups, with effect sizes in the 0.18–0.42 range. The 17 year old girls with diabetes were comparable to the normative population regarding symptoms of anxiety.

**Table 2:** Gender and norm comparison of median BDI-Y, BAI-Y, and SDQ Total Difficulties scores.

		BDI-Y					BAI-Y					SDQ Total Difficulties				
		Diabetes sample		Normative sample		M-W U test	Diabetes sample		Normative sample		M-W U test	Diabetes sample		Normative sample		M-W U test
		*n*	*Md*	*n*	*Md*	*p-value/r*	*n*	*Md*	*n*	*Md*	*p-value/r*	*n*	*Md*	*n*	*Md*	*p-value/r*
4–7 years	Girls											67	7.0 (4.0/11.0)	219	5.0 (3.0/9.0)	*0.01/0.16*
	Boys											63	9.0 (5.0/13.0)	240	7.0 (4.0/10.0)	*0.02/0.13*
	*p-value/r*												*0.16/0.12*		*0.000/0.19*	
8–10 years	Girls	73	3.0 (1.0/7.0)	235	11.0 (6.0/16.0)	*<0.001/0.39*	73	5.0 (3.0/8.5)	236	13.0 (8.0/19.0)	*<0.001/0.42*	83	5.0 (2.0/9.0)	145	5.0 (2.0/8.0)	*0.63/0.03*
	Boys	59	4.0 (2.0/8.0)	211	9.0 (4.0/14.0)	*<0.001/0.23*	59	8.0 (3.0/13.0)	208	10.0 (6.0/16.0)	*0.003/0.18*	66	9.0 (5.0/12.3)	160	6.0 (3.0/9.4)	*0.001/0.21*
	*p-value/r*		*0.22/0.11*		*0.01/0.12*			*0.17/0.12*		*0.002/0.15*			*<0.001/0.30*		*0.04/0.12*	
11–14 years	Girls	169	5.0 (2.0/12.0)	277	11.0 (7.0/16.0)	*<0.001/0.31*	168	7.0 (3.0/12.0)	273	13.0 (9.0/18.0)	*<0.001/0.40*	182	6.0 (2.0/9.0)	218	6.0 (3.0/9.0)	*0.47/0.03*
	Boys	167	3.0 (1.0/7.0)	292	8.0 (4.0/12.0)	*<0.001/0.32*	167	4.0 (2.0/9.0)	294	10.0 (6.0/14.0)	*<0.001/0.37*	185	7.0 (3.0/11.0)	205	5.0 (3.0/9.0)	*0.004/0.15*
	*p-value/r*		*0.001/0.18*		*<0.001/0.25*			*0.001/0.18*		*<0.001/0.28*			*0.004/0.15*		*0.58/0.03*	
15–16 years	Girls	125	8.0 (2.0/15.0)	128	10.0 (6.0/17.0)	*0.01/0.15*	125	8.0 (4.5/15.0)	128	12.0 (8.3/18.0)	*<0.001/0.23*	136	6.0 (2.0/10.0)	100	5.0 (2.3/9.0)	*0.55/0.04*
	Boys	111	3.0 (1.0/8.0)	104	5.0 (2.0/9.0)	*0.08/0.12*	111	5.0 (1.0/11.0)	104	9.0 (6.0/13.0)	*<0.001/0.33*	124	7.0 (3.0/11.0)	85	6.0 (3.0/9.0)	*0.12/0.11*
	*p-value/r*				*<0.001/0.38*			*<0.001/0.29*		*<0.001/0.27*			*0.16/0.09*		*0.68/0.03*	
17 years	Girls	37	11.0 (5.5/20.0)	55	10.0 (5.0/16.0)	*0.55/0.06*	37	11.0 (6.0/16.5)	55	12.0 (8.0/19.0)	*0.24/0.12*					
	Boys	45	3.0 (0.0/7.0)	44	4.5 (2.0/8.8)	*0.12/0.17*	45	4.0 (1.0/7.5)	44	7.0 (3.0/10.0)	*0.01/0.27*					
	*p-value/r*				*<0.001/0.39*			*<0.001/0.51*		*<0.001/0.43*						

*Md* =  median; *r* =  effect size. In the parantheses the 25^th^/75^th^ percentiles of the scores are provided.

Significance level; *p*<0.05.

According to caregivers' reports of social and emotional difficulties (SDQ Total difficulties, Table 2), parents generally rated younger boys (<15 years) with diabetes to experience more problems than the normative sample, with effect sizes between 0.13 and 0.21. Only young girls (4–7 years) with diabetes differed significantly from the normative sample (*r* = 0.16) regarding the Total Difficulties score.

The caregivers in the diabetes sample reported their children to experience more problems in relation to two particular SDQ problem areas compared with the norm sample: the ‘Emotional Symptoms’ and ‘Conduct Problems’ subscales (*r*: 0.10–0.31; Table 3). On the ‘Emotional Symptoms’ subscale, both girls in the diabetes sample who were 4–7 year old, and those in the 11–14 year age group, as well as boys in all age groups reportedly experienced more problems than the norm groups. Regarding the ‘Conduct Problems’ subscale, boys between the age of 8 and 14 years and young girls (4–7 years) in particular received significantly higher scores than children without diabetes.

**Table 3:** Median SDQ subscale score comparisons for diabetes and norm samples.

		4–7 Years			8–10 Years			11–14 Years			15–16 Years		
		Type 1 dia-betes	Nor-ma-tive	*p-value/r*	Type 1dia-betes	Nor-ma-tive	*p-value/r*	Type 1 dia-betes	Nor-ma-tive	*p-value/r*	Type 1 dia-betes	Nor-ma-tive	*p-value/r*
Emo-tional symptoms	Girls	2.0 (1.0/4.0)	1.0 (0.0/2.0)	*0.000/0.24*	1.0 (0.0/3.0)	1.0 (0.0/3.0)	*0.36/0.06*	2.0 (1.0/3.0)	1.25 (0.0/3.0)	*0.05/0.10*	2.0 (1.0/4.0)	2.0 (0.0/3.0)	*0.36/0.06*
	Boys	2.0 (1.0/4.0)	1.0 (0.0/2.0)	*0.000/0.22*	2.5 (1.0/4.0)	1.0 (0.0/2.0)	*0.000/0.31*	2.0 (1.0/4.0)	1.0 (0.0/2.0)	*0.000/0.24*	2.0 (0.0/3.0)	1.0 (0.0/2.0)	*0.01/0.17*
	*p-value/r*	.*95/.005*	*0.63/0.02*		*0.008/0.22*	*0.24/0.07*		*0.56/0.03*	*0.007/0.13*		*0.40/0.05*	*0.02/0.17*	
Con-duct Problems	Girls	2.0 (1.0/2.0)	1.0 (0.0/2.0)	*0.002/0.18*	1.0 (0.0/2.0)	1.0 (0.0/2.0)	.*031/0.07*	1.0 (0.0/2.0)	1.0 (0.0/2.0)	*0.55/0.03*	1.0 (0.0/2.0)	1.0 (0.0/2.0)	*0.39/0.06*
	Boys	2.0 (1.0/3.0)	1.0 (1.0/2.0)	*0.10/0.09*	2.0 (0.0/3.0)	1.0 (0.0/2.0)	*0.009/0.17*	1.0 (0.0/2.0)	1.0 (0.0/2.0)	*0.008/0.13* *	1.0 (0.0/2.0)	1.0 (0.0/2.0)	*0.53/0.04*
	*p-value/r*	*0.39/0.08*	*0.000/0.17* **		*0.04/0.17*	*0.30/0.06*		*0.02/0.12* **	*0.41/0.04*		*0.93/0.005*	*0.91/0.008*	
Hyperactivity/Inattention	Girls	2.0 (0.0/4.0)	2.0 (1.0/3.0)	*0.75/0.02*	1.0 (0.0/3.0)	1.0 (0.0/3.0)	*0.55/0.04*	1.0 (0.0/3.0)	2.0 (0.0/3.0)	*0.07/0.09*	2.0 (0.0/3.0)	2.0 (0.0/2.4)	*0.69/0.03*
	Boys	3.0 (2.0/6.0)	3.0 (1.5/5.0)	*0.46/0.04*	3.0 (1.0/5.0)	3.0 (1.0/5.0)	*0.41/0.05*	2.0 (1.0/4.0)	2.0 (1.0/4.0)	*0.38/0.04*	2.0 (1.0/5.0)	2.0 (1.0/3.5)	*0.52/0.04*
	*p-value/r*	*0.006/0.24*	*0.000/0.25*		*0.000/0.29*	*0.000/0.26*		*0.000/0.22*	*0.04/0.10* **		*0.004/0.18* **	*0.02/0.18* **	
Peer Problems	Girls	0.0 (0.0/2.0)	0.0 (0.0/1.0)	*0.22/0.07*	0.0 (0.0/1.0)	0.0 (0.0/2.0)	*0.21/0.08*	1.0 (0.0/2.0)	0.0 (0.0/2.0)	*0.13/0.08*	0.0 (0.0/2.0)	0.0 (0.0/2.0)	*0.89/0.009*
	Boys	1.0 (0.0/2.0)	0.0 (0.0/2.0)	*0.09/0.10*	1.0 (0.0/2.0)	0.0 (0.0/1.8)	*0.16/0.09*	1.0 (0.0/2.0)	1.0 (0.0/2.0)	*0.43/0.04*	1.0 (0.0/3.0)	1.0 (0.0/2.0)	*0.83/0.01*
	*p-value/r*	*0.16/0.12*	*0.03/0.10* **		*0.01/0.20* **	*0.87/0.009*		*0.04/0.11* **	*0.94/0.003*		*0.07/0.11*	*0.10/0.12*	
Prosocial Behavior	Girls	8.0 (7.0/9.0)	9.0 (7.0/10.0)	*0.20/0.08*	9.0 (8.0/10.0)	9.0 (8.5/10.0)	*0.75/0.02*	9.0 (8.0/10.0)	9.0 (8.0/10.0)	*0.02/0.11* *	9.0 (8.0/10.0)	9.0 (8.0/10.0)	*0.08/0.11*
	Boys	8.0 (7.0/9.0)	8.0 (7.0/9.0)	*0.46/0.04*	9.0 (7.0/10.0)	9.0 (8.0/10.0)	*0.77/0.02*	9.0 (7.0/10.0)	9.0 (7.0/10.0)	*0.30/0.05*	9.0 (7.0/10.0)	9.0 (7.0/10.0)	*0.75/0.02*
	*p-value/r*	*0.43/0.07*	*0.000/0.22* ***		*0.04/0.17* ***	*0.000/0.20* ***		*0.01/0.14* ***	*0.03/0.11* ***		*0.001/0.21* ***	*0.07/0.14*	

Data presented are medians, results of Mann-Whitney *U* tests (*M-W tests*), and effect sizes (*r*). In the parantheses the 25^th^/75^th^ percentiles of the scores are provided.

Significance level; *p*<0.05.

*Despite identical median scores participants from diabetes sample have the highest mean rank.

**Despite identical median scores boys have higher mean rank than girls.

***Despite identical median scores girls have higher mean rank than boys.

As observed in the norm sample, the median level of reported symptoms of depression and anxiety was higher among girls with diabetes than boys, with effect sizes between 0.18 and 0.51. The only exception was younger boys with diabetes (8–10 years), who reported their median level of symptoms of both depression and anxiety to exceed that of age-matched girls. However, this gender difference was not statistically significant.

Although the pattern of sex differences observed for SDQ Total Difficulties was less consistent among individual samples, boys tended to experience more problems. Caregivers of children and adolescents with diabetes reported that boys aged 8–10 and 11–14 years experienced significantly more problems than girls (*r: 0.15–0.30*), whereas in the normative sample, 4–7 and 8–10 year old boys were reported to experience more problems than girls (*r: 0.12–0.19*).

### Participants with elevated scores

Although particularly younger participants in the diabetes sample reported a lower median level of symptoms of depression and anxiety, the diabetes and norm samples did not differ significantly for most age/gender subgroups with regard to proportion of participants with elevated scores in relation to symptoms of depression (Table 4). Only the subgroups of 11–14 year old girls, and 11–14, and 15–16 year old boys had significantly less participants scoring in the ‘Slightly elevated’, or ‘Moderately elevated’ range. With regard to symptoms of anxiety most of the subgroups in the diabetes sample were comparable to the norm population when looking at proportion of elevated scores. The only exceptions were the groups with 8–10 year old girls, 11–14 year old girls and boys, and 15–16 year old boys that had a significantly smaller proportion of participants scoring in the ‘Slightly elevated’ or ‘Moderately elevated’ range. All of the subgroups in the diabetes sample were however comparable to the norm population regarding proportion of participants scoring in the ‘Extremely elevated’ range on both the BDI-Y and BAI-Y.

**Table 4:** Percentage of participants with elevated scores compared with normative sample.

		‘Slightly elevated’/*‘Moderately elevated’*/’Extremely elevated’ BDI-Y Scores			‘Slightly elevated’/*‘Moderately elevated’*/‘Highly elevated’ BAI-Y Scores			‘Borderline’/*‘Clinical’* SDQ Scores		
		Type 1 diabetes	Normative	*Z* test/p-value	Type 1 diabetes	Normative	*Z* test/p-value	Type 1 diabetes	Normative	*Z* test/p-value
4–7 years	Girls							6.0%	11.9%	−1.38/0.17
								*22.4%*	*6.4%*	*3.80/0.001*
	Boys							12.7%	11.3%	0.32/0.75
								*22.2%*	*7.9%*	*3.24/0.001*
8–10 years	Girls	5.5% (2.2/13.3)	12.3% (8.7/17.3)	−1.66/0.10	4.1% (1.4/11.4)	14.8% (10.9/19.9)	−2.44/0.01	14.5%	12.4%	0.44/0.66
		*4.1% (1.4/11.4)*	*11.1% (7.7/15.7)*	*−1.78/0.08*	*4.1% (1.4/11.4)*	*10.6% (7.3/15.2)*	*−1.69/0.09*	*12.0%*	*7.6%*	*1.12/0.26*
		1.4% (0.2/7.4)	3.8% (2.0/7.1)	−1.04/0.30	0%	3.8% (2.0/7.1)	−1.69/0.09			
	Boys	10.2% (4.8/20.5)	12.8% (9.0/18.0)	−0.54/0.59	5.1% (1.7/13.9)	12.0% (8.3/17.1)	−1.53/0.13	22.7%	11.9%	2.08/0.04
		*5.1% (1.7/13.9)*	*9.5% (6.2/14.2)*	*−1.07*/*0.28*	*8.5% (3.7/18.4)*	*12% (8.3/17.1)*	*−0.76/0.45*	*18.2%*	*7.5%*	*2.37/0.02*
		6.8% (2.7/16.2)	4.3% (2.3/7.9)	0.80/0.42	0%	3.4% (1.6/6.8)	−1.43/0.15			
11–14 years	Girls	5.3% (2.8/9.8)	9.4% (6.5/13.3)	−1.55/0.12	4.2% (2.0/8.4)	15.8% (11.9/20.5)	−3.73/0.0002	7.1%	9.6%	−0.89/0.37
		*6.5% (3.7/11.3)*	*13% (9.5/17.5)*	−*2.16/0.03*	*5.4% (2.9/9.9)*	*11.0% (7.8/15.3)*	−*2.02/0.04*	*9.9%*	*9.2%*	*0.24/0.81*
		5.3% (2.8/9.8)	5.4% (3.3/8.8)	−0.04/0.97	3.6% (1.7/7.6)	5.1% (3.1/8.4)	−0.76/0.45			
	Boys	3.6% (1.7/7.6)	8.6% (5.9/12.3)	−2.04/0.04	6.0% (3.3/10.7)	15.6% (11.9/20.2)	−3.05/0.002	16.8%	9.3%/	2.21/0.03
		*6.6% (3.7/11.4)*	*14.4% (10.8/18.9)*	−*2.51/0.01*	*6.0% (3.3/10.7)*	*10.2% (7.2/14.2)*	−*1.55/0.12*	*13.5%*	*7.3%*	*2.01/0.04*
		6.0% (3.3/10.7)	4.8% (2.9/7.9)	0.55/0.58	3.0% (1.3/6.8)	5.8% (3.6/9.1)	−1.35/0.17			
15–16 years	Girls	9.6% (5.6/16.0)	12.5% (7.8/19.3)	−0.74/0.46	5.6% (2.7/11.1)	8.6% (4.9/14.7)	−0.93/0.35	14.0%	9.0%	1.17/0.24
		*8.8% (5.0/15.1)*	*10.2% (6.0/16.6)*	−*0.37/0.71*	*8.0% (4.4/14.1)*	*13.3% (8.5/20.2)*	−*1.36/0.17*	*9.6%*	*9.0%*	*0.15/0.88*
		7.2% (3.8/13.1)	7.0% (3.7/12.8)	0.05/0.96	4.8% (2.2/10.1)	7.0% (3.7/12.8)	−0.75/0.45			
	Boys	7.2% (3.7/13.6)	16.3% (10.5/24.6)	−2.09/0.04	3.6% (1.4/8.9)	12.5% (7.5/20.2)	−2.42/0.02	10.5%	9.4%	0.25/0.80
		*14.4% (9.1/22.1)*	*8.7% (4.6/15.6)*	*1.32/0.19*	*12.6% (7.7/20.1)*	*13.5% (8.2/21.3)*	−*0.18/0.86*	*18.5%*	*8.2%*	*2.09/0.04*
		5.4% (2.5/11.3)	5.8% (2.7/12.0)	−0.12/0.90	3.6% (1.4/8.9)	5.8% (2.7/12.0)	−0.75/0.45			
17 years	Girls	8.1% (2.8/21.3)	12.7% (6.3/24.0)	−0.70/0.48	8.1% (2.8/21.3)	12.7% (6.3/24.0)	−0.70/0.48			
		*16.2% (7.7/31.1)*	*14.5% (7.6/26.2)*	*0.22/0.83*	*8.1% (2.8/21.3)*	*14.5% (7.6/26.2)*	−*0.93/0.35*			
		13.5% (5.6/28.0)	5.5% (1.9/14.9)	1.35/0.18	5.4% (1.5/17.7)	3.6% (1.0/12.3)	0.41/0.68			
	Boys	8.9% (3.5/20.7)	15.9% (7.9/29.4)	−1.01/0.31	4.4% (1.2/14.8)	6.8% (2.4/18.2)	−0.47/0.62			
		*11.1% (4.8/23.5)*	*11.4% (5.0/24.0)*	−*0.04/0.97*	*6.7% (2.3/17.9)*	*13.6% (6.4/26.7)*	−*1.09/0.28*			
		2.2% (0.4/11.6)	2.3% (0.4/11.8)	−0.02/0.98	0%	2.3% (0.4/11.8)	−1.02/0.31			

Significance level; *p* = 0.05. In the parentheses the upper and lower 95% confidence interval of the proportions are provided.

A significantly larger percentage of caregivers of boys with diabetes reported their child/adolescent to experience social and emotional difficulties than did caregivers of boys in the normative sample - particularly with regard to ‘Clinical’ levels of such difficulties (Table 4). Also among the caregivers of 4–7 year old girls a significantly larger proportion of respondents reported Total Difficulties scores in the ‘Clinical’ range.

### Correlations between symptoms of emotional and behavioral difficulties, and HbA_1c_, psychosocial, socio-demographic, and treatment-related variables

Table 5 summarizes the correlations between child assessment of symptoms of depression and anxiety; caregiver SDQ scores; and HbA_1c_, adherence level, and quality of life. Correlations were observed between child/adolescent reports of symptoms of depression and anxiety, and caregiver reports of SDQ Total Difficulties and all SDQ subscales, except the Prosocial Behavior subscale. Except for the strong correlation between the BDI-Y and the BAI-Y these correlations were all in the small to medium range.

**Table 5:** Correlations between outcome variable, socio-demographic, and treatment-related variables and BDI-Y, BAI-Y, and SDQ scores.

	BDI-Y	BAI-Y	SDQ Total Difficult-ties	SDQ Emotio-nal Symp-toms	SDQ Conduct Problems	SDQ Hyper-activity/Inatten-tion	SDQ Peer Problems	SDQ Prosocial Behavior
BDI-Y	-	-	0.42**	0.41**	0.27**	0.24**	0.30**	−0.08^ns^
BAI-Y	0.83**	-	0.39**	0.40**	0.22**	0.23**	0.25**	−0.05^ns^
HbA_1c_	0.16**	0.08*	0.19**	0.19**	0.22**	0.15**	0.05^ns^	−0.03^ns^
Adherence - Child	−0.24**	−0.15**	−0.38**	−0.29**	−0.34**	−0.33**	−0.18**	0.24**
Adherence – Caregiver	−0.21**	−0.13**	−0.39**	−0.29**	−0.37**	−0.33**	−0.19**	.16**
Quality of life - Child	−0.72**	−0.69**	−0.49**	−0.42**	−0.32**	−0.37**	−0.32**	0.18**
Quality of life – Caregiver	−0.43**	−0.39**	−0.74**	−0.66**	−0.51**	−0.53**	−0.49**	0.29**
Duration of diabetes	0.03	−0.01	0.01	0.003	0.05	−0.02	0.05	0.002
Use of insulin pump	−0.02	−0.02	−0.13**	−0.12**	−0.06	−0.13**	−0.08*	0.05
Other chronic disease	0.09*	0.06	0.18**	0.16**	0.08*	0.14**	0.13**	−0.03
Household income	0.02	0.04	−0.05	−0.05	−0.04	−0.03	−0.03	0.01
Caregiver respon-dent's level of education	−0.09*	−0.10*	−0.14**	−0.09**	−0.09**	−0.14**	−0.10**	0.08*

* = *p*<0.05.

** = *p*<0.01.

BDI-Y, BAI-Y, and SDQ scores correlated significantly with HbA_1c_, adherence, and quality of life, with the correlations between quality of life and nearly all subscales (except the ‘SDQ Prosocial Behavior’ subscale) being in the medium to large range (Table 5). Although the correlations between HbA_1c_ and the BDI, BAI, and SDQ Total Difficulties scores were small a comparison of children/adolescents with optimal HbA_1c_ (<7.5, *n* = 321) vs. suboptimal HbA_1c_ (≥9, *n* = 185) control showed, that the group with optimal control had significantly lower scores on the BDI-Y (*M* = 6.10, *SD* = 7.84), the BAI-Y (*M* = 7.20, *SD* = 6.91), and the SDQ (*M* = 6.42, *SD* = 5.41) than the group with suboptimal control (*M_BDI-Y_* = 10.01, *SD_BDI-Y_* = 10.13, *p*<0.001, *d* = −0.4; *M_BAI-Y_* = 9.53, *SD_BAI-Y_* = 8.50, *p* = 0.005, *d* = −0.3; *M_SDQ_* = 9.38, *SD_SDQ_* = 5.50, *p*<0.001, *d* = −0.5) indicating that the participants with suboptimal metabolic control were more emotionally or behaviorally troubled than those with optimal metabolic control.

As shown in Table 5 no significant correlations were observed between diabetes duration or household income level and depression, anxiety, SDQ Total Difficulties score, or any of the SDQ subscales. Use of insulin pump, presence of other chronic disease, and level of education of the responding caregiver were all correlated with some of the psychosocial measures investigated (see Table 5). The significant correlations found were however all small.

Looking at the possible connection between the family structure/living situation of the child/adolescent with diabetes and symptoms of depression showed, that the group of adolescents living at a continuation school (Danish boarding schools for pupils aged 14–18 years) was the only group with a significantly higher mean BDI-Y score (*Md* = 10.5, *x^2^* (df = 6)  = 13.21, *p* = 0.04), than all other groups (living with both biological parents (*Md* = 4.0); spending equal amount of time living with parents who are divorced (*Md* = 3.0); living with mom or dad who are alone (*Md* = 5.0); living with one parent and his/her new partner (*Md* = 5.0); living in foster care or treatment facility (*Md* = 5.0); or having left home to live by one self (*Md* = 22.0,)). But if applying a Bonferonni correction resulting in an adjusted alpha level of 0.008 (0.05/6), none of the between group differences reach significance. No between-group differences were found for symptoms of anxiety.

With regard to SDQ Total Difficulties scores, children/adolescents living with both biological parents had significantly better SDQ Total Difficulties median scores (*Md* = 6.0), than children/adolescents living with a single parent ((*Md* = 8.0), *U* = 25396.5, *z* = −2.265, *p* = 0.02, *r* = 0.1). Children/adolescents living in foster care or at a treatment facility had significantly worse median scores (*Md* = 13.0, *x^2^* (df = 5)  = 18.57, *p* = 0.002) than nearly all other groups; not only children living with both parents or a single parent, but also than children spending equal amount of time living with parents who are divorced (*Md* = 6.0), living with one parent and his/her new partner (*Md* = 8.0), but not those living at a continuation school (*Md* = 10.5). The significant differences were sustained even after applying the Bonferonni adjusted alpha level of 0.01 (0.05/5).

## Conclusions

The purpose of the present study was to assess the prevalence of symptoms of emotional, behavioral, and social problems in Danish children and adolescents with type 1diabetes, and to evaluate the possible associations between these symptoms and metabolic control, adherence and quality of life, as well as socio-demographic and treatment-related variables.

The self-reported median level of symptoms of depression and anxiety in this national group of pediatric diabetes patients indicates that the children with diabetes experience lower or comparable levels of symptoms compared with the Danish normative sample.

Studies have previously found children and adolescents with chronic diseases to report lower or equal levels of psychological distress compared with normative samples [11], [35]. One explanation may be that children with chronic disease (in this case diabetes) currently receive adequate medical treatment to minimize somatic symptoms and the negative consequences of living with a chronic disease, and at the same time are subject to positive attention and reinforcement from caregivers and health care personnel, which may prevent the development of symptoms of psychological distress. As mentioned previously Denmark has a public health care system that ensures equal access to medical treatment to all citizens, which means that the families in which a child is diagnosed with diabetes do not face a significant financial burden because of the disease. The public health care system also provides psychological counseling to children and adolescents with diabetes and their families if it is deemed necessary. This naturally does not prevent the stress of living with diabetes, nor perhaps the development of emotional difficulties, but it might ease the process of psychosocial adaptation. In any case the self-reported level of depression and anxiety of Danish children and adolescents with diabetes would seem not to be extremely negatively affected by living with this chronic disease.

Another explanation may be that chronically ill children have a tendency to under-report levels of psychological distress; perhaps as a result of the use a repressive style of coping in an effort to preserve their psychological well-being [36].

Methodological issues connected with the use of self-report questionnaires in this type of study could also be part of the explanation (please refer to the Limitation section below).

Further research is needed to investigate possible explanations for the our finding that Danish children and adolescents with diabetes self-report less emotional distressed than children and adolescents without diabetes, and also show less emotional difficulties than what has previously been reported in other international studies of children and adolescents with diabetes [12], [13].

Caregiver reports indicate that they perceive the social and emotional well-being of their child/adolescent with diabetes less positive. Caregivers report that boys, regardless of age, and young girls (< 8 years) with diabetes experience higher levels of various difficulties than the normative sample, while the older female age groups exhibit levels similar to the normative sample.

The individual subscales of the SDQ show that it is the ‘Emotional symptoms’ and ‘Conduct problems’ subscales that distinguish the diabetes sample from the normative sample, as median scores are higher across many of the sex and age groups. These subscale findings are in accordance with previous results demonstrating emotional and behavioral problems to be the predominant psychosocial problems in this pediatric group [12], [15].

Several possible explanations exist as to why only a moderate correlation is observed between child and caregiver reports of emotional difficulties. It may be explained by the fact that the BYI and SDQ are different instruments, and therefore may measure different aspects of the child's functioning. It is however also a consistent finding that ratings of social, emotional, or behavioral problems provided by caregivers and children often show discrepancies [37]. Low parent-child agreement has been observed across different assessment formats and measures, including structured interviews assessing mental disorders [38], and questionnaire-based assessment of quality of life [39].

Some studies have observed that when caregiver reports are compared with child self-reports of emotional distress and quality of life, there is a tendency for caregivers to report that the child experiences greater levels of psychological difficulty than is self-reported by the child [2]. One possible reason for this phenomenon may be that having a child diagnosed with a chronic disease such as diabetes cause many parents to experience a great deal of distress, and may in some cases even lead to parents developing symptoms of depression or post-traumatic stress which might affect their view of the well-being of their child [40], [41]. Others have found that parents might also be better at evaluating primarily observable behavior, while the inner lives of chronically ill children are better evaluated by the children themselves [42]. De Witt et al. (2007) [43] did however find parent and child reports on comparable questionnaires measuring psychosocial well-being to be largely concordant, but also found parents to rate their children less favorable with respect to behavioral problems, than children did themselves.

Rather than assuming that low agreement between parents and children reflects a lack of reliable judgment by one of the informants, parents and children may report uniquely different information. Neither of which should be disregarded in the evaluation of the well-being of the child.

Using highly standardized, structured interview to compare chronically ill children to healthy subject on prevalence of metal disorder, Canning [38] found, that more cases of mental disorder were identified by parents compared to child interview alone in the chronically ill group, while the opposite was the case in the group of healthy subject. Canning and colleagues have also demonstrated that reliance on a single informant (whether child or parent) lead to a failure to identify one third to one half of psychiatric disorders in a sample of chronically ill children [42]. Thus, a multi-informant approach to the evaluation of psychosocial problems in children is the best-practice approach, while also taking into consideration that rating scales are not diagnostic instruments, and should not substitute for actual diagnostic evaluation. However, rating scales are valid means of screening to evaluate the need for further psychological intervention.

Though the median level of self-reported symptoms of depression and anxiety among older girls in our sample was comparable to the norm sample, the proportion of girls in this age group scoring in the ‘Extremely elevated’ range confirms the results of previous studies indicating this group to be particularly vulnerable to developing these symptoms [11]. Our study also highlights the necessity of not disregarding the psychological difficulties of boys, particularly when looking at caregivers’ evaluations. From a clinical perspective, the detection of even minor symptoms of psychosocial problems is of interest, supporting the effort to prevent the not-uncommon continuation of psychological difficulties during the transition from adolescence to young adulthood [44], [45].

A strong correlation was observed between symptoms of depression and anxiety, and the quality of life report of the child/adolescent, just as the SDQ Total Difficulties score correlated strongly with the caregiver-reported quality of life assessment of the child. Small but significant correlations were observed between both the metabolic control and the adherence behavior of children/adolescents with diabetes, and BDI-Y, BAI-Y, and SDQ scores. However, comparing patient groups with optimal versus suboptimal metabolic control confirmed the association of HbA_1c_ with symptoms of emotional and social problems, as patients with suboptimal control scored significantly higher on the BDI-Y, BAI-Y, and SDQ.

This is not the first study only to find small but significant associations between emotional symptoms and metabolic control [9], [12]. One possible reason for the lack of a strong correlation might be the use of self-report questionnaires as a mean of assessment. A meta-analysis of studies that examined the association between depression and metabolic control among adult patients with type 1 diabetes observed a stronger correlation between these variables in studies based on standardized interviews and diagnostic criteria than in studies based on self-reporting [46]. Another reason may be the participation rate, as discussed in the Limitations section below.

While living with a single parent only appeared to influence caregiver assessment of emotional and behavioral difficulties, children and adolescents living at either continuation schools, in foster care, or at a treatment facility might need special attention with regard to symptoms of emotional and behavioral problems.

### Limitations

This study has certain limitations. First, 48.1% of the children and adolescents with diabetes who were invited to participate chose not to take part in the study. We know that this group had a significantly higher HbA_1c_, but we do not know their psychological well-being. This study, as others before it [6], [9], [12], [45], found symptoms of emotional problems to be associated with higher HbA_1c_ level. Also symptoms of depression and anxiety might influence energy level, and may therefore influence the inclination to participate in a survey such as this. Still we can only speculate whether the inclusion of data from this group might have led to an increase in the level of reported symptoms.

A second limitation is the relatively small samples used for subgroup analyses.

Thirdly the length of the survey in which the questionnaire assessing symptoms of depression and anxiety was included was rather extensive. As the BYI subscales were at the end of the survey, attrition might have caused participants to be less nuanced in their responses, in order to finish the survey more quickly. Furthermore the BYI has no reversed questions, which might have made it possible to test whether the respondents were actually reading the questions and actively relating to their content. However, a strong correlation was observed between symptoms of depression and anxiety and the child's self-reported quality of life, a finding that supports the validity of the child/adolescent self-report, and is consistent with previous results [47].

Another limitation is the lack of an objective assessment of emotional and behavioral difficulties in this pediatric group. The study would have benefitted from the addition of clinical, diagnostic interviews.

Despite these limitations, this study should be acknowledged for the large, national population samples involved allowing sub group comparisons. Additionally, the wide age range of the participating children and adolescents distinguishes this study from many of the preceding studies concerning the emotional and social well-being of children and adolescents with type 1 diabetes.

### Final remarks

In summary, this study finds that Danish children and adolescents with type 1 diabetes report lower or comparable median levels of symptoms of depression and anxiety than their age-matched peers, while the numbers of patients with elevated scores are generally similar to the normative sample.

While the older girls in this study did not differ significantly from the normative sample, the high proportion of 17 year old girls with diabetes scoring in the ‘Extremely elevated’ range indicates a certain vulnerability in this group that should not be ignored. With regard to the caregivers of children and adolescents with diabetes, this study finds that they report boys and young girls to experience more difficulties than the normative sample, particularly with regard to ‘Conduct problems’. Caregivers also report nearly all age groups in the diabetes sample to experience more emotional symptoms compared to the normative sample, and report the prevalence of scores in the ‘clinical’ range for boys and young girls to exceed the prevalence in the normative group.

Since the general Danish pediatric diabetes population appears to be reasonably emotionally well-adjusted, the results of this study do not support the necessity of screening the entire pediatric diabetes population for symptoms of social and emotional difficulties. However, significant associations between emotional symptoms and metabolic control were found. Whether symptoms of emotional distress leads to poor adherence and suboptimal metabolic control, or poor metabolic control leads to symptoms of emotional distress is yet to be determined.

Further studies are needed to assess whether multi-informant assessment of psychosocial difficulties might prove beneficent in reaching a new understanding of the barriers to optimal metabolic control that some of these patients are facing.
